# Compounds That Have an Anti-Biofilm Effect against Common Bacteria at Very Low Concentrations and Their Antibiotic Combination Effect

**DOI:** 10.3390/antibiotics12050853

**Published:** 2023-05-05

**Authors:** Hyeon-Ji Hwang, Dan-dan Li, Jieun Lee, Min Kyung Kang, Hyung Ryong Moon, Joon-Hee Lee

**Affiliations:** 1 College of Pharmacy, Pusan National University, Busan 46241, Republic of Korea; hyunjee0822@gmail.com (H.-J.H.); dany0930@naver.com (D.-d.L.); yijiun@pusan.ac.kr (J.L.); kmk87106@pusan.ac.kr (M.K.K.); mhr108@pusan.ac.kr (H.R.M.); 2 Research Institute for Drug Development, Pusan National University, Busan 46241, Republic of Korea

**Keywords:** biofilm, anti-biofilm compound, synergy, *Escherichia coli*, *Bacillus subtilis*, *Staphylococcus aureus*, *Pseudomonas aeruginosa*, *Salmonella enterica*

## Abstract

Two synthetic compounds, MHY1383, azo-resveratrol and MHY1387, 5-[4-hydroxy-3,5-methoxybenzy]-2-thioxodihydropyrimidine-4,6[1H,5H]-dione have been reported to have an anti-biofilm effect on *Pseudomonas aeruginosa* at very low concentrations (1–10 pM). Here, we investigated the anti-biofilm effects of these compounds in various bacteria. We found that MHY1383 significantly inhibited *Escherichia coli*, *Bacillus subtilis*, and *Staphylococcus aureus* biofilm formation at 1 pM, 1 nM, and 10 nM, respectively. MHY1387 also inhibited the biofilm formation of *E. coli*, *B. subtilis*, and *S. aureus* at 1 pM, 10 nM, and 100 pM, respectively. Both MHY1383 and MHY1387 showed medium-dependent anti-biofilm effects on *Salmonella enterica* at high concentrations (10 μM). We also tested the susceptibility to antibiotics by measuring the minimum inhibitory concentration (MIC) in various bacteria. When *P. aeruginosa*, *E. coli*, *B. subtilis*, *S. enterica*, and *S. aureus* were treated with MHY1383 or MHY1387 in combination with four different antibiotics, the MICs of carbenicillin against *B. subtilis* and *S. aureus* were lowered more than two-fold by the combination with MHY1387. However, in all other combinations, the MIC changed within two-fold. The results of this study suggest that MHY1383 and MHY1387 are effective anti-biofilm agents and can be used at very low concentrations against biofilms formed by various types of bacteria. We also suggest that even if a substance that inhibits biofilm is used together with antibiotics, it does not necessarily have the effect of lowering the MIC of the antibiotics.

## 1. Introduction

Biofilms are recalcitrant to antibiotic therapy and a major cause of persistent and recurrent infections by clinically important pathogens [1,2,3]. A large number of diseases are associated with biofilms formed by pathogens. According to the US National Institutes of Health, it is estimated that biofilms account for over 80% of microbial infections in the human body [4]. In addition, biofilms cause harmful biofouling, surface erosion, and pathogenic contamination in the food and pharmaceutical industries [1,5,6]. Therefore, developing anti-biofilm agents is of great importance both in the medical field and in industry.

**Figure 1:**
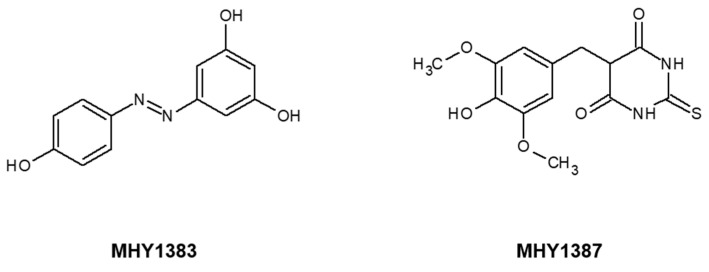
Molecular structure of MHY1383 (azo-resveratrol) and MHY1387 (5-[4-hydroxy-3,5-methoxybenzy]-2-thioxodihydropyrimidine-4,6[1H,5H]-dione) [9].

Anti-biofilm agents can be potentially obtained from either natural products or synthetic compounds [7], and there are several categorized sources for anti-biofilm activity, such as quorum sensing (QS) inhibitors, electro-chemicals, antimicrobial peptides or lipids, phytochemicals, and nickel nanoparticles [2,6,8]. Among them, QS, the bacterial cell-to-cell communication mechanism, is very important for bacterial biofilm formation and has been recognized as a major target for obtaining anti-biofilm activity [3]. Recently, we discovered two promising anti-biofilm compounds, MHY1383 and MHY1387, from the screening of anti-QS compounds [9]. MHY1383, azo-resveratrol, and MHY1387, 5-[4-hydroxy-3,5-methoxybenzy]-2-thioxodihydropyrimidine-4,6[1H,5H]-dione (Figure 1), had anti-QS, anti-virulence, and anti-biofilm activities against *Pseudomonas aeruginosa* [9]. These two compounds have been previously reported for different activities: MHY1383 has an inhibitory effect on mushroom tyrosinase, and MHY1387 inhibits the production of reactive oxygen species and peroxynitrite in vitro [10,11]. Probably due to this antioxidant activity, MHY1387 was reported to suppress the lipopolysaccharide-induced nuclear factor kappa B activation and protein expression of cyclooxygenase 2 and inducible nitric oxide synthase in macrophages [11].

In addition to these useful activities, these compounds showed promising activity in inhibiting biofilm formation at extremely low concentrations (1 to 10 pM) [9]. This means that they have a useful activity that can be used in combination with antibiotics or developed as anti-pathogens that specifically inhibit only the pathogenicity of pathogens. However, in previous studies, the anti-biofilm activity of these compounds was intensively studied only against *P. aeruginosa*. Therefore, in this study, we addressed the effects of MHY1383 and MHY1387 on biofilm formation by various other bacteria, such as *Escherichia coli*, *Bacillus subtilis*, *Salmonella enterica*, and *Staphylococcus aureus*. We found that both MHY1383 and MHY1387 have the anti-biofilm effect on a broad spectrum of bacteria, suggesting that these compounds are promising candidates for the development of anti-biofilm agents.

## 2. Results

### 2.1. MHY1383 Had Anti-Biofilm Effects on Common Bacteria at Very Low Concentrations

**Figure 2:**
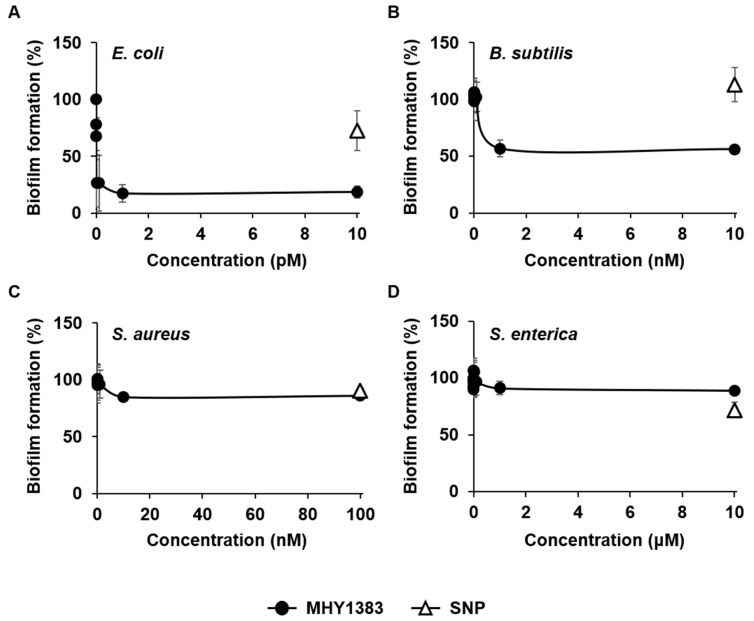
Biofilm inhibitory effect of MHY1383. *E. coli* (**A**), *B. subtilis* (**B**), *S. aureus* (**C**), and *S. enterica* (**D**) were treated with various concentrations of MHY1383. Biofilm formations were quantified by static biofilm assay. Data were presented relative to the sample without an inhibitor (which corresponds to 100%). For comparison, the degree of inhibition by 5 μM SNP is indicated as open triangles in panels.

For this study, we selected four bacterial species, *E. coli*, *B. subtilis*, *S. enterica* serovar Typhimurium, and *S. aureus*. These strains cover both Gram-positive and negative bacteria commonly found and that include important pathogenic genera [12]. We investigated the effect of MHY1383 on biofilm formation by these bacteria in a static biofilm system. Biofilm formations of *E. coli* and *B. subtilis* were effectively inhibited by 83% and 43% at 1 pM and 1 nM, respectively (Figure 2A,B). The biofilm formation of *S. aureus* was also significantly inhibited, but to a lesser extent (15%) at higher concentrations (10 nM) (Figure 2C). In the case of *S. enterica*, biofilm formation was only inhibited to a very small extent (9%) at much higher concentrations (1 μM) (Figure 2D). In all bacterial species, the effect of MHY1383 plateaued at the concentrations mentioned above, and treatment with higher concentrations did not further inhibit biofilm formation.

Our previous study has shown that MHY1383 inhibited the biofilm formation of *P. aeruginosa* in a very similar manner: the biofilm formation was almost maximally inhibited by 1 pM MHY1383, and at the concentrations higher than 1 pM, the inhibition plateaued [9]. However, the strength of the effect and the concentration that showed the effect differed depending on the bacterial species. In the case of *E. coli*, the concentration of MHY1383 exhibiting the maximum effect was 1 pM, which was similar to that of *P. aeruginosa*, but biofilm formation was inhibited much more effectively at that concentration (Figure 2A). In the case of *B. subtilis,* the inhibitory concentration was 1000 times higher than that of *P. aeruginosa*, but the degree of inhibition was similar (Figure 2B). In *S. aureus* and *S. enterica*, both the concentration and degree of inhibition of biofilm formation were not as good as those of *P. aeruginosa* (Figure 2C,D).

Although the concentration of MHY1383 that inhibits the biofilm of *B. subtilis*, *S. aureus*, and *S. enterica* is higher than that of *P. aeruginosa*, it is still very low compared to other biofilm inhibitors. For comparison, each bacterium was treated with sodium nitroprusside (SNP), a well-known biofilm inhibitor [13], at a concentration of 5 μM under the same conditions, and how much biofilm formation was inhibited was investigated. The SNP reduced the biofilm formation of *E. coli*, *S. aureus*, and *S. enterica* by 27%, 10%, and 28%, respectively (Figure 2A,C,D). SNP was not able to inhibit the biofilm formation of *B. subtilis*, whereas MHY1383 could inhibit it (Figure 2B). These results demonstrate that the biofilm inhibitory effect of MHY1383 is better than SNP or at least comparable. The only exception was *S. enterica*, for which SNP showed a biofilm inhibition effect of 28%, whereas MHY1383 showed a very limited biofilm inhibition effect of 9%. However, the concentration showing this level of inhibitory effect was 1 μM for MHY1383, which was much lower than 5 μM for SNP.

Summarizing these results, MHY1383 has a better biofilm inhibitory effect compared to SNP, showing much lower effective concentrations and a higher degree of inhibition to *E. coli*, *B. subtilis*, and *S. aureus* than SNP (Figure 2A–C).

### 2.2. MHY1387 Also Effectively Inhibited the Biofilm Formation of a Wide Range of Bacteria at Very Low Concentrations

**Figure 3:**
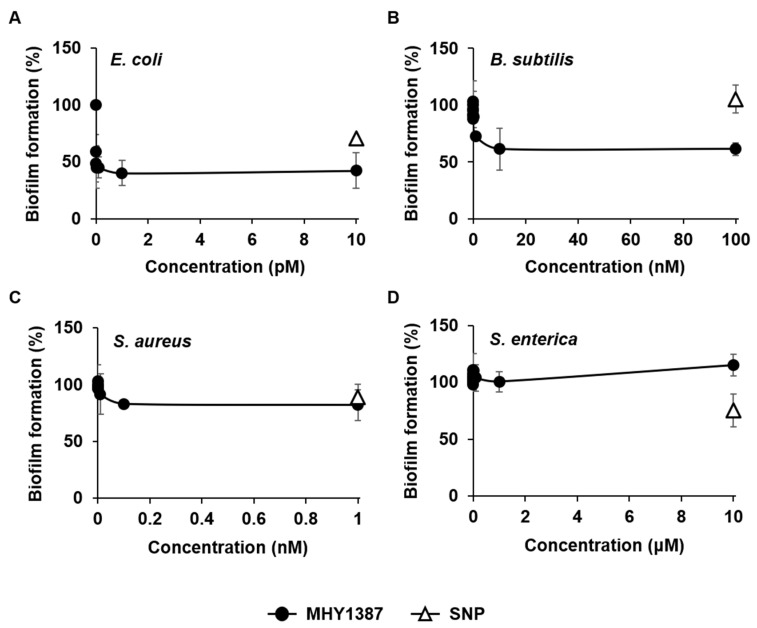
Biofilm inhibitory effect of MHY1387. *E. coli* (**A**), *B. subtilis* (**B**), *S. aureus* (**C**), and *S. enterica* (**D**) were treated with various concentrations of MHY1387, and biofilm formations were quantified by static biofilm assay in the same manner as in Figure 2. Data were presented relative to the sample without an inhibitor, and the degree of inhibition by 5 μM SNP was indicated as open triangles for comparison.

Next, we performed the same experiment with MHY1387. Like MHY1383, MHY1387 also profoundly inhibited the biofilm formation of *E. coli* and *B. subtilis* by 60% and 39% at 1 pM and 10 nM, respectively (Figure 3A,B). The effect on *E. coli* was similar to that of MHY1383 in terms of the degree of inhibition and the effective concentration (Figure 2A), but for *B. subtilis*, MHY1387 showed a similar inhibitory effect at a concentration about 10 times higher than that of MHY1383 (Figure 2B). MHY1387 also inhibited the biofilm formation of *S. aureus* by 17% at 100 pM, which is a similar degree of inhibition to MHY1383, but the inhibitory concentration is about 100 times lower (Figure 3C). Like MHY1383, the inhibition plateaued at higher concentrations. The biofilm of *S. enterica* was not inhibited even at 10 μM (Figure 3D).

When comparing the activity of MHY1387 with 5 μM SNP, MHY1387 showed a much better effect in both the degree of biofilm inhibition and the effective concentration in the case of *E. coli*, *B. subtilis*, and *S. aureus*, showing much lower effective concentrations than that of SNP (Figure 3A–C). Like MHY1383, MHY1387 failed to inhibit *S. enterica* biofilm formation, whereas SNPs could (Figure 3D).

MHY1383 and MHY1387 showed similar effects on the type of bacteria and degree of biofilm inhibition, but against *B. subtilis*, MHY1383 showed an effect at a concentration about 10 times lower, and against *S. aureus*, MHY1387 showed an effect at a concentration about 100 times lower. All these results indicate that both MHY1383 and MHY1387 effectively inhibit the biofilm formation of various bacteria at very low concentrations.

### 2.3. The Biofilm Inhibitory Effect of MHY1383 and MHY1387 on S. enterica May Vary Depending on the Medium

**Figure 4:**
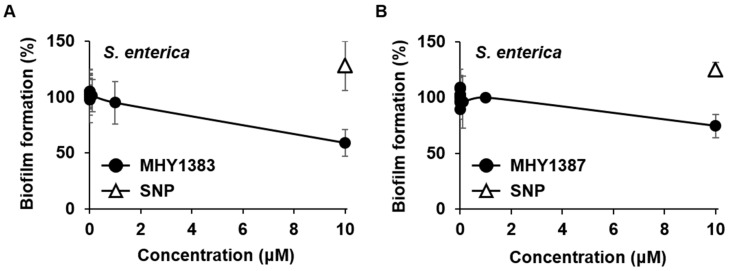
Effects of MHY1383 and MHY1387 on biofilm formation of *S. enterica* in different media. In Figure 2 and Figure 3, *S. enterica* was treated with MHY1383 and MHY1387 in LB medium, whereas in this experiment, *S. enterica* was treated with different concentrations of MHY1383 (**A**) and MHY1387 (**B**) in M63 minimal medium containing 0.5% casamino acid and 0.2% glucose. All experiments were performed in the same manner as in Figure 2 and Figure 3, and the degree of inhibition by 5 μM SNP was also indicated as open triangles for comparison.

MHY1383 and MHY1387 showed good effects against various bacteria but poor effects against *S. enterica*. However, our previous study showed that MHY1387 and SNP had different biofilm inhibitory effects depending on the medium [9]. Therefore, we investigated whether the inhibitory effect of MHY1383 and MHY1387 on *S. enterica* biofilm could be seen in other media. To this end, instead of the LB used in the previous experiments, the same biofilm formation experiment was performed in M63 minimal medium containing glucose and casamino acid as carbon sources. The results showed that both MHY1383 and MHY1387 inhibited the biofilm formation of *S. enterica* by 41% and 25%, respectively, at a high concentration of 10 μM (Figure 4A,B). Interestingly, in this medium, SNP did not show any biofilm inhibitory effect (Figure 4A,B). This result indicates that MHY1383 and MHY1387 can have a better biofilm inhibitory effect than SNP depending on the medium for *S. enterica*, although it appears at high concentrations. Taken together, it can be concluded that MHY1383 and MHY1387 work effectively against a wide range of bacterial species.

### 2.4. MHY1383 and MHY1387 Mostly Little Affect the Minimum Inhibitory Concentration (MIC) of Antibiotics against Bacteria

Since MHY1383 and MHY1387 significantly inhibit the biofilm formation of bacteria, we investigated whether the MIC of antibiotics against bacteria could be lowered in the presence of MHY1383 and MHY1387. Since our previous study had already shown that MHY1383 and MHY1387 effectively inhibit biofilm formation of *P. aeruginosa* at very low concentrations [9], in this experiment, we included *P. aeruginosa* and investigated the MIC of four different antibiotics, carbenicillin, gentamicin, tobramycin, and polymyxin B. To sum it up, two MHYs were co-treated with these four different antibiotics at 100 pM representing low concentration and 5 μM representing high concentration to five bacterial species, *P. aeruginosa*, *E. coli*, *B. subtilis*, *S. aureus*, and *S. enterica*. In all these cases, the MIC was measured.

**Figure 5:**
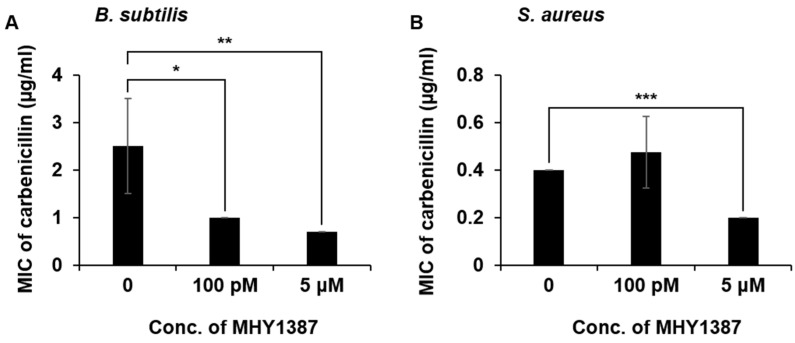
Decrease in minimum inhibitory concentration (MIC) of carbenicillin in the presence of MHY1387. MIC of carbenicillin to *B. subtilis* (**A**) and *S. aureus* (**B**) was determined in the presence of MHY1387 (100 pM or 5 μM), as described in the Materials and Methods. *, *p* < 0.05; **, *p* < 0.01; ***, *p* < 0.005.

In the case of MHY1383, there was no case where the MIC was lowered more than two-fold when used in combination with antibiotics, while there were a few cases where the MIC was reduced more or less by less than two-fold (Table 1). However, in the case of MHY1387, the MIC of carbenicillin against *B. subtilis* and *S. aureus* was lowered more than two-fold (Figure 5A,B). In particular, for *B. subtilis*, even when MHY1387 was co-treated with carbenicillin at a low concentration of 100 pM, the MIC of carbenicillin was reduced 2.5-fold (Figure 5A). For *S. aureus*, the MIC of carbenicillin was reduced two-fold when MHY1387 was treated at 5 μM (Figure 5B). Like MHY1383, there were a few more cases where the MIC was reduced more or less by less than two-fold (Table 2).

However, overall, there was no noticeable change in MIC even when MHYs were treated together, except for the case of MHY1387 and carbenicillin mentioned above. We will discuss this below.

## 3. Discussion

Our previous study reported that MHY1383 and MHY1387 have both anti-QS and anti-biofilm activities in *P. aeruginosa* at very low concentrations: anti-QS effect at 100 pM and anti-biofilm effect at 1–10 pM [9]. In this study, we further investigated the effects of MHY1383 and MHY1387 on biofilm formation of various bacterial species and newly found the followings: (1) MHY1383 and MHY1387 have broad-spectrum anti-biofilm activities against *E. coli*, *B. subtilis*, and *S. aureus*. (2) These compounds reduce biofilm formation of these bacteria at picomolar to nanomolar concentrations. (3) In *E. coli* and *B. subtilis*, biofilm formation was better inhibited by MHY1383 than MHY1387, whereas MHY1387 was able to more effectively inhibit the biofilm formation of *S. aureus*. (4) Both MHY1383 and MHY1387 have limited anti-biofilm effect on *S. enterica* at high concentration (10 μM) depending on the medium. (5) The combined treatment of antibiotics and MHYs did not significantly change the MICs of antibiotics as a whole, but in the case of carbenicillin, the MICs for *B. subtilis* and *S. aureus* were lowered more than two-fold.

Since the strains used in this study represent common bacterial species frequently found in everyday life, the broad-spectrum anti-biofilm activity of MHY1383 and MHY1387 is a significant advantage as an anti-biofilm agent. In addition, MHY1383 and MHY1387 work at extremely low concentrations in the range of 1 pM to 10 nM. This advantage is also very important. Well-known biofilm inhibitors, SNP and anthranilate, inhibited biofilm at 5 μM and 100 μM, respectively [13,14]. Our two MHYs showed similar or much better effects at much lower concentrations than these. These low concentrations of drugs will be helpful in minimizing the potential toxic side effects. It has already been confirmed that MHY1387 is not cytotoxic at a concentration of up to 50 μM [11].

Among the findings from this study, the following deserves discussion. First, despite inhibiting bacterial biofilm formation, MHY1383 and MHY1387 did not lower the MIC of antibiotics in many cases when used in combination with antibiotics. At first glance, this does not seem to fit well with the general idea that bacterial biofilm formation significantly increases resistance to antibiotics. However, recently, the concept of antibiotic resistance has been divided into resistance, tolerance, and persistence in more detail, and it has been argued that the reason why biofilms can protect cells from antibiotics is because of an increase in tolerance or persistence rather than an increase in resistance [15,16]. Conventionally, antibiotic ‘resistance’ is used to describe the ability of microbes to grow in the presence of an elevated level of an antibiotic and quantified by the MIC [15]. In contrast, ‘tolerance’ and ‘persistence’ mean the ability of microbes to survive transient exposure to high concentrations of an antibiotic without a change in the MIC [15]. When we say that the biofilm is resistant to antibiotics, it emphasizes that the bacterial cells in the biofilm can survive more persistently in the presence of antibiotics [16,17]. Therefore, biofilm cells actually have tolerance rather than resistance to antibiotics, and therefore, inhibition of biofilm formation is also targeted at tolerance, so the MIC may not have changed. In fact, the MIC did not change even when anthranilate, which has a biofilm inhibitory effect, was treated in combination with antibiotics [18].

Second, MHY1383 and MHY1387 showed different effects on the biofilm formation of *S. enterica*, depending on the media. Many studies showed that the nutritional conditions in the environment can influence the formation, development, and dispersion of biofilm, so the biofilm inhibitor activity can depend on the media composition and initial cell density [5,19,20]. The different anti-biofilm effects of MHY1383 and MHY1387, depending on media composition, are also considered to be one of these examples.

Third, SNP shows no effects on the biofilm formation of *B. subtilis*. SNP is a nitrogen oxide (NO) donor, and NO has been known to induce biofilm dispersion in several bacterial species, including *P. aeruginosa*, *S. aureus*, and *E. coli* [13,21,22]. However, it has been reported that the effect of NO on biofilm formation appears differently in some bacterial species: NO was found to enhance the biofilm formation of *Vibrio harveyi* and *Shewanella oneidensis* [23,24] and had no effects on the biofilm formation of *B. subtilis* [25].

The findings in this study highlight the potency of MHY1383 and MHY1387 against the biofilm formation of various bacteria. The properties of MHYs are very unique in that they exhibit anti-biofilm effects on various types of bacteria at extremely low concentrations. Thus, we suggest that MHY1383 and MHT1387 are promising candidate compounds to combat bacterial biofilm-mediated infections. The general mechanism by which MHY1383 and MHY1387 exert their inhibitory effects on biofilm formation against multiple species of bacteria remains unclear. In our previous study [9], we found that both MHY1383 and MHY1387 inhibit biofilm formation by reducing intracellular signaling molecule, 3′,5′-cyclic diguanylate (c-di-GMP), in *P. aeruginosa*. However, while c-di-GMP plays an important role in controlling biofilm formation in many Gram-negative bacteria [5,13,26], it has not yet been confirmed whether MHY1383 and MHY1387 lower the intracellular c-di-GMP in other bacterial species than *P. aeruginosa*. In addition, in the case of *S. aureus*, it is known that a messenger molecule other than c-di-GMP is used to regulate biofilm formation [27,28]. Therefore, further studies are needed to elucidate the underlying general mechanism.

## 4. Materials and Methods

### 4.1. Bacterial Strains and Culture Conditions

The bacterial strains used in this study are listed in Table 3. *E. coli*, *B. subtilis*, *S. enterica*, *S. aureus*, and *P. aeruginosa* strains were mostly grown in Luria-Bertani (LB; tryptone 10 g/L, yeast extract 5 g/L, and NaCl 5 g/L) medium at 37 °C with vigorous shaking. Bacterial growth was measured using the optical density at 600 nm (OD_600_). MHY1383 and MHY1387 were dissolved in dimethyl sulfoxide (DMSO) and diluted to the indicated concentrations in the media.

### 4.2. Biofilm Assay

Biofilm analyses were performed in the following media; 1/4-diluted LB supplemented with 0.1% mannitol for *E. coli*, and TSB medium (30 g/L of Bacto^TM^ Tryptic Soy Broth, BD, Baltimore, MD, USA) supplemented with 0.5% glucose for *B. subtilis*, and LB for *S. aureus* [12,31]. For *S. enterica*, we used two different media: LB for the experiments in Figure 2 and Figure 3, and M63 minimal medium (M63 salt [KH_2_PO_4_, 3 g/L; K_2_HPO_4_, 7 g/L; (NH_4_)_2_SO_4_, 2 g/L], 1 mM MgSO_4_), supplemented with 0.5% casamino acid and 0.2% glucose for the experiment in Figure 4. The biofilms were quantified using a static biofilm assay, as described previously, with slight modification [12]. Overnight cultures of cells were inoculated at OD_600_ = 0.06 into fresh media on a 96-well polystyrene plate. MHY1383 and MHY1387 were added at the indicated concentrations into these media, and the cells were grown at 30 °C for 48 h without shaking. After the cell growth was measured by OD_600_, planktonic cells were poured out, and the plate was washed with water and dried for 2 min. Then, 180 μL of crystal violet (0.1%, wt/vol) was added to each well and incubated for 10 min to stain the biofilms attached to the well surface. After a brief wash, the biofilm-staining crystal violet was dissolved in 200 μL of 30% acetic acid. The biofilm formation was quantified from the staining levels measured by absorbance at 600 nm (A_600_), which was normalized by cell growth (OD_600_). The data were presented relative to the sample without an inhibitor (which corresponds to 100%).

### 4.3. Bacterial Susceptibility to Antibiotics (MIC Measurement)

Overnight cultures of the *P. aeruginosa*, *E. coli*, *B. subtilis*, *S. enterica*, and *S. aureus* strains were diluted to OD_600_ = 0.0001 in LB medium on 96-well plates. Two concentrations (100 pM and 5 μM) of MHY1383 or MHY1387 and serial dilutions of 4 different antibiotics (carbenicillin, gentamicin, tobramycin, and polymyxin B) were added into each well containing bacterial cells in all combinations and incubated at 37 °C for 24 h. MIC was evaluated from the cell growth on Sensititre™ Manual Viewbox (Thermo Scientific™, Waltham, MA, USA). The concentration ranges of the serial dilutions of antibiotics used for MIC measurement are shown in Table 4.

### 4.4. Statistical Analysis

The Student’s *t*-test (two-sample assuming equal variances) was used to determine the significance of differences using Microsoft Office Excel 2019. *p* < 0.05 was considered significant. All of the experiments were carried out in triplicate and repeated at least twice independently.

## 5. Conclusions

Two synthetic compounds, MHY1383, azo-resveratrol and MHY1387, 5-[4-hydroxy-3,5-methoxybenzy]-2-thioxodihydropyrimidine-4,6[1H,5H]-dione, which have been previously reported to have an anti-biofilm effect on *P. aeruginosa* at very low concentrations (1–10 pM), were investigated for their anti-biofilm effects on various bacteria. Both MHY1383 and MHY1387 inhibited the biofilm formation by *E. coli*, *B. subtilis*, and *S. aureus* at very low concentrations of 1 pM–10 nM. Together with the previous results from *P. aeruginosa*, this result indicates that the anti-biofilm effect of these two compounds at very low concentrations is a common effect for various bacteria. However, when these two compounds were used together with several antibiotics against various bacteria, the effect of lowering the MIC of the antibiotics was not generally observed. MHY1387 significantly lowered the MIC when used together with carbenicillin against *B. subtilis* and *S. aureus*, while the MIC of the antibiotic was lowered only within two-fold in other combinations.

## Figures and Tables

**Table 1:** Minimum inhibitory concentration (MIC) of carbenicillin, gentamicin, tobramycin, and polymyxin B in combination with MHY1383 against various bacteria.

	MIC (μg/mL)			
Strains	Antibiotics	MIC Alone	MIC in Combination with 100 pM MHY1383	MIC in Combination with 5 μM MHY1383
*P. aeruginosa*	Carbenicillin	13.67 ± 1.53	14.67 ± 0.58	13.67 ± 1.53
	Gentamicin	2.88 ± 0.48	2.63 ± 0.25	2.63 ± 0.85
	Tobramycin	3.25 ± 0.50	3.38 ± 0.63	3.25 ± 0.50
	Polymyxin B	3.50 ± 1.00	4.13 ± 1.31	3.00 ± 0.00
*E. coli*	Carbenicillin	4.00 ± 0.00	4.00 ± 0.00	4.00 ± 0.00
	Gentamicin	5.00 ± 1.00	6.00 ± 2.00	4.50 ± 1.32
	Tobramycin	8.00 ± 0.00	8.33 ± 0.58	8.00 ± 1.00
	Polymyxin B	0.83 ± 0.05	0.88 ± 0.10	0.75 ± 0.10
*B. subtilis*	Carbenicillin	2.25 ± 1.77	2.00 ± 1.41	1.60 ± 1.27
	Gentamicin	3.83 ± 1.15	4.17 ± 1.44	4.00 ± 1.32
	Tobramycin	3.63 ± 1.25	3.75 ± 1.19	3.75 ± 1.50
	Polymyxin B	15.00 ± 0.00	15.00 ± 0.00	18.33 ± 5.77
*S. enterica*	Carbenicillin	4.00 ± 0.00	3.50 ± 1.00	4.00 ± 0.00
	Gentamicin	9.50 ± 1.00	9.50 ± 1.00	9.00 ± 1.63
	Tobramycin	14.75 ± 2.50	14.75 ± 2.50	13.75 ± 2.06
	Polymyxin B	3.00 ± 0.00	3.00 ± 0.50	2.83 ± 0.29
*S. aureus*	Carbenicillin	1.50 ± 0.71	1.35 ± 0.92	1.00 ± 0.00
	Gentamicin	5.50 ± 0.00	5.50 ± 0.00	5.50 ± 0.00
	Tobramycin	11.00 ± 0.00	10.67 ± 0.58	11.00 ± 0.00
	Polymyxin B	350.00 ± 0.00	400.00 ± 50.00	350.00 ± 50.00

Data are means ± SD of four independent experiments. SD, standard deviation; MIC, minimal inhibitory concentration.

**Table 2:** MIC of carbenicillin, gentamicin, tobramycin, and polymyxin B in combination with MHY1387 against various bacteria.

	MIC (μg/mL)			
Strains	Antibiotics	MIC Alone	MIC in Combination with 100 pM MHY1387	MIC in Combination with 5 μM MHY1387
*P. aeruginosa*	Carbenicillin	10.00 ± 0.00	12.00 ± 0.00	10.00 ± 0.00
	Gentamicin	2.50 ± 0.00	2.63 ± 0.25	2.13 ± 0.25
	Tobramycin	3.50 ± 0.00	3.13 ± 0.25	3.50 ± 0.00
	Polymyxin B	5.13 ± 0.25	5.00 ± 0.00	4.13 ± 0.25
*E. coli*	Carbenicillin	6.50 ± 1.00	6.50 ± 1.00	6.50 ± 1.00
	Gentamicin	6.00 ± 0.00	6.00 ± 0.00	6.00 ± 0.00
	Tobramycin	10.00 ± 0.00	10.25 ± 0.50	10.25 ± 0.50
	Polymyxin B	1.00 ± 0.00	1.03 ± 0.05	1.03 ± 0.05
*B. subtilis*	Carbenicillin	2.50 ± 1.00	1.00 ± 0.00	0.70 ± 0.00
	Gentamicin	3.63 ± 0.25	3.50 ± 0.00	4.00 ± 0.00
	Tobramycin	4.13 ± 0.25	4.00 ± 0.00	4.13 ± 0.25
	Polymyxin B	20.00 ± 0.00	20.00 ± 0.00	20.00 ± 0.00
*S. enterica*	Carbenicillin	4.00 ± 0.00	4.00 ± 0.00	4.00 ± 0.00
	Gentamicin	5.25 ± 0.50	5.00 ± 0.00	5.00 ± 0.00
	Tobramycin	12.00 ± 0.00	9.25 ± 0.50	9.25 ± 0.50
	Polymyxin B	3.63 ± 0.25	4.50 ± 0.00	2.50 ± 0.00
*S. aureus*	Carbenicillin	0.40 ± 0.00	0.48 ± 0.15	0.20 ± 0.00
	Gentamicin	4.00 ± 0.00	4.63 ± 0.25	3.50 ± 0.00
	Tobramycin	6.00 ± 0.00	5.25 ± 0.50	5.25 ± 0.50
	Polymyxin B	212.50 ± 25.00	212.50 ± 25.00	350.00 ± 0.00

Data are means ± SD of four independent experiments. SD, standard deviation; MIC, minimal inhibitory concentration.

**Table 3:** Bacterial strains used in this study.

Name	Genotype	References
*Escherichia coli*		
MG1655	A wild type strain of *E. coli* K-12	Lab. collection
*Bacillus subtilis*		
ATCC6051	A wild type strain of *B. subtilis*	Lab. collection
*Salmonella enterica*		
SL1344	A type of *S. enterica* serovar Typhimurium	Lab. collection
*Staphylococcus aureus*		
RN4220	A wild type strain of *S. aureus*	[29]
*Pseudomonas aeruginosa*		
PAO1	A wild type strain of *P. aeruginosa*	[30]

**Table 4:** Antibiotic concentration ranges for MIC assays.

	The Concentration Range for MIC Assay (μg/mL)			
Strains/Antibiotics	Carbenicillin	Gentamicin	Tobramycin	Polymyxin B
*P. aeruginosa*	0.2–14	0.5–5.5	0.5–5.5	0.5–5.5
*E. coli*		0.5–5.5	1–11	0.1–1.1
*B. subtilis*		0.5–5.5	0.5–5.5	5–55
*S. enterica*		1–11	1–11	0.5–5.5
*S. aureus*		0.5–5.5	1–11	50–550

## Data Availability

Not applicable.
